# The association between systemic lupus erythematosus and dementia A meta-analysis

**DOI:** 10.1590/1980-57642018dn12-020006

**Published:** 2018

**Authors:** Zhuoxian Zhao, Natalia P. Rocha, Haitham Salem, Breno S. Diniz, Antonio L. Teixeira

**Affiliations:** 1 MD. Neuropsychiatry Program, Department of Psychiatry and Behavioral Sciences, McGovern Medical School, The University of Texas Health Science Center at Houston. 1941 East Road, Houston, TX 77054, USA.; 2 PhD. Neuropsychiatry Program, Department of Psychiatry and Behavioral Sciences, McGovern Medical School, The University of Texas Health Science Center at Houston. 1941 East Road, Houston, TX 77054, USA.; 3 MD. Neuropsychiatry Program, Department of Psychiatry and Behavioral Sciences, McGovern Medical School, The University of Texas Health Science Center at Houston. 1941 East Road, Houston, TX 77054, USA. Neuropsychiatry Program, Department of Psychiatry and Behavioral Sciences, McGovern Medical School, The University of Texas Health Science Center at Houston. 1941 East Road, Houston, TX 77054, USA.; 4 MD, PhD. Harris County Psychiatric Center, McGovern Medical School, The University of Texas Health Science Center at Houston, 2800 S MacGregor Way, Houston, TX 77021, USA.; 5 MD, PhD. Neuropsychiatry Program, Department of Psychiatry and Behavioral Sciences, McGovern Medical School, The University of Texas Health Science Center at Houston. 1941 East Road, Houston, TX 77054, USA. Harris County Psychiatric Center, McGovern Medical School, The University of Texas Health Science Center at Houston, 2800 S MacGregor Way, Houston, TX 77021, USA.; The University of Texas Health Science Center at Houston, McGovern Medical School, Harris County Psychiatric Center, S MacGregor Way, Houston, USA

**Keywords:** systemic lupus erythematosus, antiphospholipid antibodies, dementia, cognitive dysfunction, lúpus eritematoso sistêmico, anticorpos antifosfolípides, demência, disfunção cognitiva

## Abstract

A growing body of evidence indicates that systemic lupus erythematosus (SLE) is associated with increased risk of cognitive impairment and dementia. However, to date, no studies have been conducted to quantitatively summarize and evaluate the consistency of data.

**Objective:**

To quantitatively evaluate the relationship of SLE and antiphospholipid antibodies (aPL) with cognitive dysfunction and dementia.

**Methods:**

All relevant literature was retrieved from Pubmed, Scopus, and PsycINFO databases. The meta-analysis was performed using effect estimates and 95% confidence intervals (CIs) to calculate pooled risk estimates. The heterogeneity among studies was also examined.

**Results:**

The meta-analysis included 11 original studies involving a total of 81,668 patients with dementia and 407 patients with cognitive dysfunction. There were significant associations on fixed-effect models between SLE and dementia (3 studies; RR=1.50; 95% CI=1.37-1.64), SLE and cognitive dysfunction (4 studies; OR=2.97; 95% CI=1.72-5.15), and aPL and cognitive dysfunction (5 studies, OR=1.97; 95% CI=1.55-2.52). We also combined cognitive dysfunction and dementia outcomes as they both represented cognitive impairment. There were significant associations between aPL and cognitive impairment (6 studies; OR=2.03; 95% CI=1.62-2.55), and SLE and cognitive impairment (7 studies; OR=1.83; 95% CI=1.42-2.35). Moderate heterogeneity (I^2^=45.7%) was found in the association between SLE and cognitive impairment, low heterogeneity (I^2^=21.8%) in the association between SLE and dementia, and near zero heterogeneity for the other three main analyses.

**Conclusion:**

Both SLE and aPL are associated with cognitive impairment.

## Resumo

Um volume crescente de evidências indica que o lúpus eritematoso sistêmico (LES) está associado ao aumento do risco de comprometimento cognitivo e demência. No entanto, até o presente momento nenhum estudo foi conduzido a fim de resumir e avaliar quantitativamente a consistência dos dados.

**Objetivo:**

Avaliar quantitativamente a relação entre o LES e anticorpos anticorpos antifosfolípides (aPL) com disfunção cognitiva e demência.

**Métodos:**

Toda literatura relevante foi recuperada das bases de dados Pubmed, Scopus e PsycINFO. A meta-análise foi realizada utilizando as estimativas de efeito e os intervalos de confiança de 95% (ICs) para calcular as estimativas de risco combinadas. A heterogeneidade entre os estudos também foi examinada.

**Resultados:**

A meta-análise incluiu 11 estudos originais com um total de 81.668 pacientes com demência e 407 pacientes com disfunção cognitiva. Houve associações significativas (modelos de efeitos fixos) entre LES e demência (3 estudos, RR=1,50; IC 95%=1,37-1,64), LES e disfunção cognitiva (4 estudos; OR=2,97; IC 95%=1,72-5,15), e aPL e disfunção cognitiva (5 estudos, OR=1,97; IC 95%=1,55-2,52). Além disso, combinamos os resultados da disfunção cognitiva e demência, uma vez que ambos representaram déficit cognitivo. Houve associações significativas entre aPL e comprometimento cognitivo (6 estudos, OR=2,03, IC 95%=1,62-2,55), e LES e comprometimento cognitivo (7 estudos, OR=1,83; IC 95%=1,42-2,35). Uma heterogeneidade moderada (I2=45,7%) foi encontrada na associação entre LES e comprometimento cognitivo, heterogeneidade baixa (I2=21,8%) na associação entre LES e demência e heterogeneidade quase zero para as outras três principais análises.

**Conclusão:**

Tanto o LES como aPL estão associados a déficit cognitivo.

Systemic Lupus Erythematosus (SLE) is a severe autoimmune disease whose broad etiology involves genetic, epigenetic, hormonal and immune regulatory factors. The clinical course as well as SLE-related damage is unpredictable. Many organs may be affected, including the brain, where this may cause neuropsychiatric symptoms.^1^^,^ 2 Cognitive dysfunction was identified by the American College of Rheumatology in 1999 as a specific neuropsychiatric symptom associated with SLE, characterized by impaired cognitive processes such as attention, memory, language and problem solving.^3^

Approximately 50% of SLE patients are positive for antiphospholipid antibodies (aPL), which are composed of lupus anticoagulant (LA), anti-cardiolipin (aCL) and anti-β2GP1 antibodies.^4^ aPL are recognized as strong risk factors for hypercoagulability, microinfarction in cerebral small vessels, cerebral ischemia and thromboembolic infarctions, which are closely related to cognitive decline and ultimately to the occurrence of dementia.^5^ In addition to autoantibodies, increased tau-protein and decreased b-amyloid levels have been reported in the cerebrospinal fluid (CSF) samples of SLE patients, indicating a pathogenesis similar to Alzheimer’s disease.^6^

Several epidemiological studies have explored the associations between SLE and dementia, and of aPL with cognitive dysfunction or dementia.^5^^,^ 7 ^,^ 8 Two longitudinal cohort studies in large populations with long-term follow-ups provided strong evidence that baseline SLE was significantly associated with increased risk of dementia.^7^^,^ 8 Recently, a big data study showed that the frequency of dementia was higher among 4,886 SLE patients than in 24,430 age-frequency- and sex-frequency-matched controls without SLE.^9^ Despite the evidence of association between SLE and dementia, to date, no meta-analysis has been conducted to quantitatively summarize and evaluate the consistency of data. Furthermore, original studies have been conducted to analyze the outcome of overall neuropsychiatric manifestations in SLE,^10^ but not to specifically address cognitive dysfunction or dementia in patients with SLE or with antiphospholipid syndrome (APS).

To the best of our knowledge, there are only non-systematic literature reviews or qualitative analytical descriptions on the association between SLE and cognitive dysfunction or dementia. Hence, our study primarily aimed to quantitatively analyze the association of SLE and/or aPL with cognitive dysfunction or dementia. In order to address our research goals, data was analyzed as follows: [1] we analyzed the combined results for the associations between SLE and dementia, SLE and cognitive dysfunction, and aPL and cognitive dysfunction; [2] the combined results were examined by sensitivity analyses, and further analyzed using stratified analyses and meta-regression; in order to validate the main results, we analyzed the results under different subgroups and explored the sources of heterogeneity; [3] publication bias and the likelihood of reverse causation was also evaluated.

## METHODS

### Search strategy

A manual literature search was performed independently by two investigators (ZZ, ALT) in June 2017. Our primary search database was PubMed and the reference lists of selected articles were fully examined. Supplementary searches were then performed on the Scopus and PsycINFO databases. For the search strategy, several combinations of key words were compared, and finally the search terms that extensively covered the published articles in the research topic were chosen: (“Lupus” or “antiphospholipid” or “anticardiolipin” or “lupus anticoagulant” or “β2-glycoprotein-I” or “anti-Ro” or “anti-La”) and (“Dementia” or “Alzheimer disease” or “Vascular Dementia” or “Multi-Infarct Dementia” or “Cognitive decline” or “Cognitive impairment” or “Mild Cognitive Impairment”). The examined articles had to have been published with at least an abstract. Duplication of articles was considered, and articles with the largest number of cases were selected. The meta-analysis was conducted according to the Meta-analysis of Observational Studies in Epidemiology (MOOSE) guidelines.^11^

### Inclusion and exclusion criteria

The articles that met the following criteria were included in the meta-analysis: [1] study designs were observational studies, including prospective or retrospective cohort, case-control and cross-sectional studies; [2] the exposures of interest were the diagnosis of SLE, aPL including the subtypes of LA, aCL and anti-β2GP1 antibodies; [3] the outcome of interest was the prevalence or incidence of cognitive dysfunction or dementia; [4] the crude or adjusted results were extracted when the articles provided the effect estimates that analyzed the relationship of SLE or aPL with cognitive dysfunction or dementia; [5] if the effect estimates and 95% confidence intervals (CIs) were not provided in the articles, the raw data available in the articles were used to calculate the odds ratio (OR) or relative risk (RR) with the corresponding 95% CI; [6] authors were contacted when the articles might potentially have the results of interest but did not show them in the papers.

Since the outcome of this meta-analysis was cognitive dysfunction or dementia, studies with outcomes focusing only on other neuropsychiatric SLE manifestations (e.g. stroke, seizures, psychosis etc.) were excluded. Articles on literature reviews, *in vitro* or animal studies, case reports, and studies not analyzing the associations of interest or irrelevant studies were also excluded.

### Data extraction

Data was extracted independently by two researchers (ZZ, ALT) using a standardized extraction form. The results were further checked by another investigator (HS). First authors or corresponding authors were contacted to collect potential relevant data when the effect estimates and 95% CIs were not provided in the papers but might exist in the raw data. The following data was extracted: first author, publication year, country, study design, number of cases and controls, definition or measuring methods of cognitive dysfunction and dementia, effect estimates and 95% CIs, and the adjustments of covariates.

### Statistical analyses

Statistical analyses were performed using the STATA v14.0 software (Stata-Corp, College Station, TX, USA). A two-tailed p<0.05 was considered statistically significant. The heterogeneity between studies was assessed using the Q and I^2^ statistics. The heterogeneity test seeks to determine whether there are genuine differences underlying the results of the studies (heterogeneity), or whether the variation in findings is compatible with chance alone (homogeneity).^12^ In case of significant heterogeneity between studies, a random-effects model was used to calculate the pooled estimate and corresponding 95% CI; otherwise, a fixed-effects model was performed. For the sub-analyses, we performed: [1] sensitivity analyses by omitting one study at a time and recalculating the pooled effect estimates; [2] stratified analyses to separately analyze the pooled effect estimates and 95% CIs in the subgroups of different study designs (cross-sectional, case-control or cohort) and number of cases (>50 or <50); [3] meta-regression to analyze the effect of individual and multiple covariates on the pooled results and to explore potential sources of heterogeneity. Publication bias was examined for each of the pooled associations by visual inspection of funnel plots, Begg’s rank correlation and Egger weighted regression method. A p value <0.05 was considered significant evidence of publication bias; and a p value greater than but near 0.05 was considered possible publication bias. The Duval & Tweedie trim and fill procedure was utilized to recalculate the new effect estimate and 95% CI when publication bias was suspected.^13^

## RESULTS

### Search process

**Figure 1:**
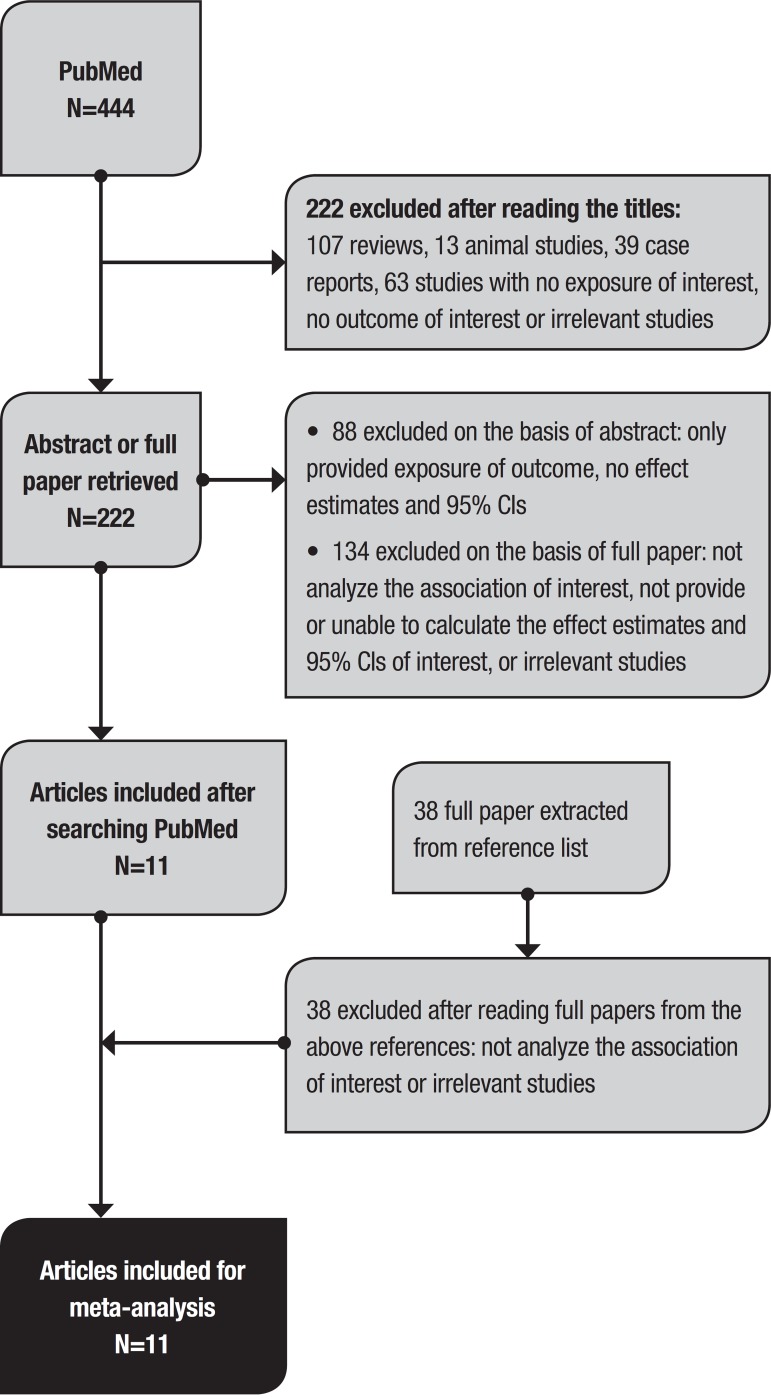
Literature search strategy.

The literature search strategy is shown in Figure 1. Articles were screened step by step: reading of the titles of articles to exclude reviews, *in vitro* or animal studies, case reports and studies that were obviously irrelevant to our topic; then abstracts or full papers were read to exclude articles without exposure or outcome of interest or unable to establish the associations of interest. Potential articles of interest were further explored from references lists.

The initial search identified 444 articles and abstracts, of which 222 were excluded by manual screening of the titles. A further 88 articles were excluded after reading the abstract. We then included 38 papers extracted from references lists. The PsycINFO database yielded no eligible papers for our topic. In total, 172 papers were reviewed in full. Eleven articles were finally included,^4^^,^ 5 ^,^ 7 ^,^ 8 ^,^ 10 ^,^ 14 ^-^ 19 of which 7 studies analyzed cognitive dysfunction as the outcome, and 4 studies had dementia as the outcome. The detailed characteristics of the 11 studies included in this meta-analysis are presented in Table 1.

**Table 1:** Characteristics of the included studies (N=11) for the associations between (SLE or aPL) and (cognitive dysfunction or dementia).

First author, publication year Study design		Country		Definition of cognitive dysfunction or dementia		Cognitive dysfunction/non-cognitive dysfunction		Comparison and effect estimates (95% CI)			Adjustment of covariates
								Cognitive dysfunction			
Tektonidou^4^, 2006Cross-sectional		Greece		3-hour, non-computerized battery of 9 neuropsychological tests		25/35		OR: SLE-APS: OR: aCL IgG: OR: aCL IgM: OR: LA:	1.45 (0.50, 4.25) 1.92 (0.34, 10.78) 0.63 (0.22, 1.78) 2.38 (0.76, 7.40)		Univariate analysis
Denburg^14^, 1997Cross-sectional		Canada		Neuropsychological assessments		118/35		OR: LA:	1.92 (1.17, 3.16)		Univariate analysis
Tomietto^15^, 2007Case-control		Italy		ACR neuropsychological assessments		36/3635/17		OR: SLE: OR: aPL:	4.4 (1.4, 14) 4.94 (1.2, 20.3)		Univariate analysis
Sanna^10^, 2003Case-control		UK, Italy		ACR neuropsychological assessments		35/288		OR: aPL: OR: aCL IgG: OR: aCL IgM: OR: LA:	2.24 (1.10-4.56) 2.18 (1.04-4.59) 0.60 (0.13-2.68) 2.43 (1.15-5.13)		Univariate analysis
Glanz^18^, 1997Case-control		Canada		2-hour neuropsychological assessments		34/71		OR: SLE:	3.20 (1.31-7.82)		Crude analysis
Hanly^19^, 1994Case-control		Canada		Standardized neuropsychological tests		17/101		OR: SLE:	5.14 (1.12-23.53)		Crude analysis
Murray^5^, 2012Prospective cohort		US		HVLT-R, COWAT		107/587		OR: aPL:	2.10 (1.3-3.41)		Gender, education, poverty status, depression, disease-related variables, cardiovascular risk-factors and events
								Dementia			
Lin^7^, 2016Retrospective cohort		Taiwan		ICD-9		77/6367		HR: SLE	2.14 (1.26-3.63)		Age and sex matched
Juby^17^, 1998Prospective cohort		Canada		Mini-Mental State Examination		37/264		RR: aCL	2.49 (1.31-4.73)		Crude analysis
Sundquist^16^, 2008Prospective cohort		Sweden		ICD8-10		Cases: 52		SIR: SLE: men SIR:SLE: women	1.45 (0.72-2.60) 1.73 (1.24-2.35)		Matched for age, sex and other factors
Wotton^8^, 2017Retrospective cohort		UK		ICD9-10		81502/1752325		RR: SLE	1.46 (1.32-1.61)		Sex, age, time period, region of residence, deprivation score

ICD: International Classification of Diseases; SLE: Systemic lupus erythematosus; aPL: antiphospholipid antibodies; APS: antiphospholipid syndrome; LA: Lupus anticoagulant; aCL: anti-cardiolipin; ACR: American College of Rheumatology; OR: odds ratio; RR: relative risk; HR: hazard ratio; SIR: standardized incidence ratio; CI: confidence interval; HVLT-R: Hopkins Verbal Learning Test – Revised; COWAT: Controlled Oral Word Association Test.

### Study characteristics

Data were extracted from the original articles and analyzed in several combinations to provide a comprehensive understanding for all the associations of interest (Table 2). Four studies provided data analyzing the association between SLE and dementia (81,631 dementia patients); 4 studies were utilized for the association between SLE and cognitive dysfunction (112 patients with cognitive dysfunction and 243 controls); 5 studies analyzed the association between aPL and cognitive dysfunction (320 patients with cognitive dysfunction and 962 controls); 6 studies analyzed the association between SLE and cognitive dysfunction, dementia (81,743 patients); and 6 studies explored the association between aPL and cognitive dysfunction, dementia (357 patients and 1,226 controls).

**Table 2:** Summary of the main meta-analysis for the associations between (SLE or aPL) and (Cognitive dysfunction or Dementia).

Meta-analysis of all combinations of interest	No. of studies	Pooled effect estimates	Heterogeneity	Publication bias
SLE and Dementia	3	RR: 1.50 (95% CI: 1.37-1.64)	Q=2.56, P=0.278, I^2^=21.8%	Begg: P=0.117; Egger: P=0.067
SLE and Cognitive dysfunction	4	OR: 2.97 (95% CI: 1.72-5.15)	Q=2.70, P=0.441, I^2^=0.0%	Begg: P=0.497; Egger: P=0.624
aPL and Cognitive dysfunction	5	OR: 1.97 (95% CI: 1.55-2.52)	Q=3.31, P=0.507, I^2^=0.0%	Begg: P=0.629; Egger: P=1.000
**Cognitive dysfunction and dementia were tentatively combined as the outcome**				
SLE and (Cognitive dysfunction or Dementia)	7	OR: 1.83 (95% CI: 1.42-2.35)	Q=11.06, P=0.087, I^2^=45.7%	Begg: P=0.099; Egger: P=0.008
aPL and (Cognitive dysfunction or Dementia)	6	OR: 2.03 (95% CI: 1.62-2.55)	Q=3.76, P=0.585, I^2^=0.0%	Begg: P=0.573;

Egger: P=0.492 SLE: systemic lupus erythematosus; aPL: antiphospholipid antibodies; OR: odds ratio; RR: relative risk; CI: confidence interval.

In summary, 11 studies were included in the meta-analysis, of which two were cross-sectional, 4 case-control, and 5 prospective or retrospective cohort studies. Four studies provided adjusted or matched data, while 7 studies provided results with only crude or univariate analysis relevant to our research purpose. There were 5 studies analyzing data with >50 cases, and most other studies involved 30-40 cases. Among the 11 studies, 19 data analyses were performed in this meta-analysis, which analyzed the exposures of SLE and aPL (aCL IgG, aCL IgM, LA) in relation with the outcomes of cognitive dysfunction and dementia.

### Main analysis and heterogeneity

For the association between SLE and dementia (3 studies),^7^^,^ 8 ^,^ 16 the combined RR was 1.50 (95% CI: 1.37-1.64) using a fixed-effect model with low heterogeneity (Q=2.56, P=0.278, I^2^=21.8%) (Table 2). As expected, the pooled effect estimate (4 studies)^4^^,^ 15 ^,^ 18 ^,^ 19 for the association between SLE and cognitive dysfunction was greater [OR: 2.97 (95% CI: 1.72-5.15)] using a fixed-effect model, with near zero heterogeneity (Q=2.70, P=0.441, I^2^=0.0%). For the association between aPL and cognitive dysfunction (5 studies),^4^^,^ 5 ^,^ 10 ^,^ 14 ^,^ 15 the pooled ORs were statistically significant [OR=1.97 (95% CI: 1.55-2.52)] with near zero heterogeneity (I^2^=0.0%).

**Figure 2:**
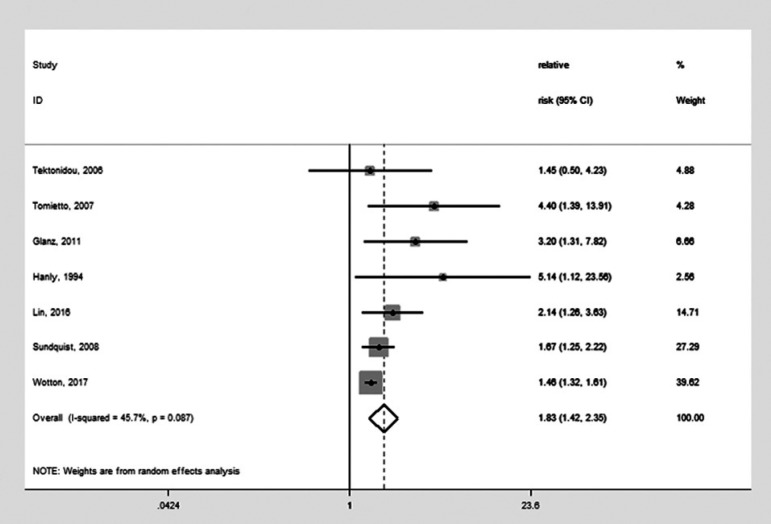
Forest plot for meta-analysis of the studies investigating systemic lupus erythematosus and dementia or cognitive dysfunction (Odds Ratio=1.83).

**Figure 3:**
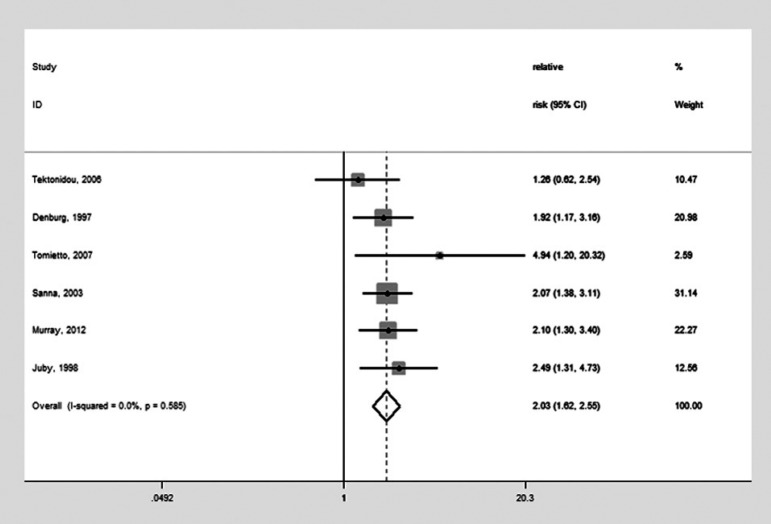
Forest plot for meta-analysis of the studies addressing the presence of antiphospholipid antibodies and cognitive dysfunction or dementia (Odds Ratio=2.03)

We also tentatively combined the outcomes of cognitive dysfunction and dementia together, given they are both part of the spectrum of cognitive impairment. Combining the 7 studies (SLE and dementia, SLE and cognitive dysfunction) (Figure 2)^4^^,^ 7 ^,^ 8 ^,^ 15 ^,^ 16 ^,^ 18 ^,^ 19 yielded an intermediate result [OR: 1.83 (95% CI: 1.42-2.35)] using a random-effect model with moderate heterogeneity (Q=11.06, P=0.087, I^2^=45.7%). When the 5 studies addressing aPL and cognitive dysfunction were combined with the study on aPL and dementia, the total 6 studies (Figure 3)^4^^,^ 5 ^,^ 10 ^,^ 14 ^,^ 15 ^,^ 17 yielded very similar pooled results [OR=1.97 (95% CI: 1.55-2.52); OR=2.03 (95% CI: 1.62-2.55)] and near zero heterogeneity (I^2^=0.0%).

### Sensitivity analyses, subgroup analyses and meta-regression

Sensitivity analyses were performed to: [1] analyze the influence of individual studies on the pooled effect estimates, [2] validate the statistically significant results, [3] identify potential sources of heterogeneity. All the 5 sub-analyses for the associations between (SLE or aPL) and (cognitive dysfunction or dementia) were statistically significant even for the smallest pooled effect estimate by the sensitivity analyses (Table 3). Omitting one article^8^ decreased the low heterogeneity (I^2^=21.8%) to almost zero in the combined association between SLE and dementia; it also decreased the moderate heterogeneity (I^2^=45.7%) to low heterogeneity (I^2^=19.3%) in the combined association between SLE and (Cognitive dysfunction or Dementia).

**Table 3:** Sensitivity and Stratified (Subgroup) analyses for the associations between (SLE or aPL) and (Cognitive dysfunction or Dementia).

Sensitivity analyses	Pooled effect estimates and 95% CI					Heterogeneity		
	Smallest		Largest			Smallest		Largest
SLE and Dementia	RR: 1.48 (95% CI: 1.35-1.63)		RR: 1.77 (95% CI: 1.37-2.27)			Q=0.65, P=0.419, I^2^=0.0%		Q=1.94, P=0.164, I^2^=48.4%
SLE and Cognitive dysfunction	OR: 2.65 (95% CI: 1.42-4.94)		OR: 3.84 (95% CI: 2.02-7.29)			Q=0.35, P=0.838, I^2^=0.0%		Q=2.66, P=0.265, I^2^=24.7%
SLE and (Cognitive dysfunction or Dementia)	OR: 1.70 (95% CI: 1.37-2.11)		OR: 2.12 (95% CI: 1.41-3.18)			Q=6.19, P=0.288, I^2^=19.3%		Q=11.05, P=0.050, I^2^=54.7%
aPL and Cognitive dysfunction	OR: 1.92 (95% CI: 1.50-2.46)		OR: 2.10 (95% CI: 1.62-2.72)			Q=1.54, P=0.674, I^2^=0.0%		Q=3.30, P=0.348, I^2^=9.1%
aPL and (Cognitive dysfunction or Dementia)	OR: 1.97 (95% CI: 1.55-2.52)		OR: 2.15 (95% CI: 1.69-2.73)			Q=1.77, P=0.777, I^2^=0.0%		Q=3.74, P=0.442, I^2^=9.1%
**Stratified (Subgroup) analyses**		**Number of studies**		**Pooled effect estimates and 95% CI**				**Heterogeneity**
SLE and Cognitive dysfunction	Case-control	3		OR: 3.84 (95% CI: 2.02-7.29)				Q=0.35, P=0.838, I^2^=0.0%
aPL and Cognitive dysfunction	Cross-sectional	2		OR: 1.67 (95% CI: 1.11-2.50)				Q=0.92, P=0.337, I^2^=0.0%
	Case-control	2		OR: 2.21 (95% CI: 1.50-3.27)				Q=1.34, P=0.246, I^2^=25.6%
	Cases>50	2		OR: 2.01 (95% CI: 1.42-2.84)				Q=0.06, P=0.800, I^2^=0.0%
	Cases<50	3		OR: 1.94 (95% CI: 1.38-2.73)				Q=3.23, P=0.199, I^2^=38.0%
aPL and (Cognitive dysfunction or Dementia)	Cases<50	4		OR: 2.05 (95% CI: 1.51-2.77)				Q=3.69, P=0.297, I^2^=18.6%
	Cohort	2		OR: 2.23 (95% CI: 1.52-3.28)				Q=0.17, P=0.678, I^2^=0.0%

SLE: Systemic lupus erythematosus; aPL: antiphospholipid antibodies; OR: odds ratio; RR: relative risk; CI: confidence interval.

*Stratified analyses (Table 3)*. For the association between SLE and cognitive dysfunction, the 3 case-control studies^15^^,^ 18 ^,^ 19 provided a higher combined result [OR: 3.84 (95% CI: 2.02-7.29)] than the results of the 4 studies (3 case-control, 1 cross-sectional) combined as the main analysis [OR: 2.97 (95% CI: 1.72-5.15)]. Case-control studies also yielded a higher pooled effect estimate (OR: 2.21) than cross-sectional studies (OR: 1.67) for the association between aPL and cognitive dysfunction, while the studies with >50 cases (I^2^=0.0%) had much lower heterogeneity than the studies with<50 cases (I^2^=38.0%). For the association between aPL and cognitive impairment, the 4 studies^4^^,^ 10 ^,^ 15 ^,^ 17 with <50 cases had higher heterogeneity (I^2^=18.6%) than the heterogeneity of the 2 cohort studies^5^^,^ 17 (I^2^=0.0%) or the 6 studies combined (I^2^=0.0%). All the subgroup analyses revealed statistically significant associations (Table 3).

*Meta-regression.* The heterogeneity in the association between SLE and dementia (I^2^=21.8%) was eliminated (residual I^2^=0%) by the covariate of number of cases which ranged from 52 to 81,502. For the relationship between SLE and dementia and cognitive dysfunction combined, the moderate heterogeneity (I^2^=45.7%) was partially reduced (residual I^2^=19.25%) by the covariate of number of cases; it was substantially reduced by study design (longitudinal *vs.* non-longitudinal), types of outcomes (dementia *vs.* cognitive dysfunction) and adjustments (adjusted *vs.* not adjusted), all with residual I^2^=4.87%, since the three covariates were considered as collinearity in the regression model; when the number of cases and another covariate (study design, types of outcomes or adjustments) were utilized together as multiple covariates, the heterogeneity was eliminated (residual I^2^=0%).

### Publication bias (Table 2)

No evidence of publication bias was suggested regarding the association between SLE and cognitive dysfunction (Begg: P=0.497; Egger: P=0.624), or the association between aPL and cognitive dysfunction (Begg: P=0.629; Egger: P=1.000). Publication bias was suspected for the association between SLE and dementia (Funnel plot was slightly asymmetric; Begg: P=0.117; Egger: P=0.067). With the trim and fill procedure, there was no significant change in the pooled result (adjusted OR=1.46, 95% CI: 1.34-1.59; number of imputed studies=2). Possible publication bias was also observed for the association between SLE and (cognitive dysfunction and dementia combined) (Funnel plot was slightly asymmetric; Begg: P=0.099; Egger: P=0.008). However, the trim and fill procedure showed no significant change in the pooled effect estimate (adjusted OR=1.60, 95% CI: 1.20-2.13; number of imputed studies=3).

## DISCUSSION

To our knowledge, this was the first meta-analysis that quantitatively analyzed the pooled data for the relationships of SLE and aPL with cognitive dysfunction or dementia. Our meta-analysis suggested significant associations between SLE and dementia (81,631 dementia patients in 3 cohort studies), SLE and cognitive dysfunction (112 patients with cognitive dysfunction in 4 case-control or cross-sectional studies), aPL and cognitive dysfunction (320 patients with cognitive dysfunction in 5 case-control or cross-sectional studies). Tentatively, cognitive dysfunction and dementia were combined as they were both part of the spectrum of cognitive impairment. There were significant associations between SLE and cognitive impairment (81,743 patients with cognitive impairment in 7 combined studies) and between aPL and cognitive impairment (357 patients with cognitive impairment in 6 combined studies). The results of all the five main analyses were consistent with the results of sensitivity analyses.

Since the exposure of this meta-analysis focused on SLE and aPL (LA, aCL and anti-β2GP1), the information was extracted from a large amount of mixed data especially on the search of different antibodies, which increased the difficulty of embarking on a meta-analysis of this topic. We designed the meta-analysis that screened so many antibodies by designing the keywords, and extracted the key data to perform the data synthesis. We also fully explored potential papers that did not directly provide the effect estimates but whose results could be calculated from the raw data, and this meta-analysis covered all the available data. Adequate sub-analyses (sensitivity analyses, subgroup analyses and meta-regression) were performed in order to validate the pooled results, analyze the data in different subgroups and explore the sources of heterogeneity. All the five main analyses were confirmed by sensitivity analyses. Subgroup analyses yielded statistically significant results as main analyses; and meta-regression reasonably explained the sources of heterogeneity (reducing heterogeneity to residual I^2^=0%). The prevalence of cognitive impairment was reported to range widely from 14 to 90% in SLE,^20^ and the domains of cognitive dysfunction in neuropsychiatric SLE also varied significantly (general intelligence, verbal learning, visuospatial skills, psychomotor dexterity and attention),^21^ rendering the comparison of the available studies complex. It is worth mentioning that co-morbidities such as depression can influence cognitive functioning. In our meta-analysis, all the included studies provided clear definitions of the outcomes, with the cognitive dysfunction measured by comprehensive neuropsychological assessments. The low between-studies heterogeneity also made measurement error unlikely. Secondly, although SLE and aPL both increased the incidence of dementia and the prevalence of cognitive dysfunction, our meta-analysis did not necessarily indicate that SLE increased dementia or cognitive dysfunction due to the aPL in patients with SLE. Regarding the underlying mechanisms, we could only assert that SLE and aPL share common biological links for the impairment of cognitive function based on evidence from epidemiological studies. Moreover, the studies with the outcome of cognitive dysfunction were all cross-sectional or case-control studies, making it impossible to rule out reverse causation. Considering the fact that the pooled association between SLE and dementia was also statistically significant and that dementia is regarded as a more severe cognitive impairment, the significant results with cognitive dysfunction were reasonable.

Autoantibodies, including aPL, in SLE are likely to attack vascular endothelial cells, activate the inflammatory response and coagulation cascade, which results in occlusive thrombosis or chronic subclinical thrombosis leading to the progressive compromise of neural activity and, hence, decline in cognitive function and, ultimately, vascular dementia.^22^ Indeed aPL have been recognized as a prominent risk factor for neuropsychiatric manifestations of SLE, including seizures and stroke, probably through this pro-thrombotic effect and direct damage to neuronal cells.^23^ Besides thrombotic phenomena, the central nervous system can be the target for autoantibodies, that can impair neuronal function and activate an inflammatory response, including the release of pro-inflammatory cytokines, chemokines and adhesion molecules.^24^^-^ 26 The activation of endothelial cells by the autoantibodies and cytokines might also damage the integrity of the blood-brain barrier, resulting in an essential step for the subsequent damage of neurons and, hence, the decline in cognitive function.^26^ In addition, increased CSF levels of tau protein - the microtubule-associated protein that reflects neuronal damage and axonal degeneration in Alzheimer’s disease - have also been described in SLE patients.^6^ Studies have also suggested significantly decreased levels of toxic metabolic products in the CSF of SLE patients, such as amyloid precursor protein and b-amyloid protein, which indicates increased deposition and accumulation in the brain.^6^ Dedicated studies are necessary to investigate the possible multiple mechanisms underlying the association between cognitive impairment and SLE.

In summary, this meta-analysis suggested that patients with SLE and the presence of aPL were both positively associated with cognitive dysfunction and dementia.
